# Severe Dengue Associated With Hemophagocytic Lymphohistiocytosis (HLH) in an Adult ICU

**DOI:** 10.7759/cureus.82443

**Published:** 2025-04-17

**Authors:** Jitendra S Chahar, Meru V Kumar, Afzal Azim, Banani Poddar, Mohan Gurjar, Dinesh Chandra

**Affiliations:** 1 Critical Care Medicine, Sanjay Gandhi Postgraduate Institute of Medical Sciences, Lucknow, IND; 2 Hematology, Sanjay Gandhi Postgraduate Institute of Medical Sciences, Lucknow, IND

**Keywords:** dengue-associated hemophagocytic lymphohistiocytosis (hlh), h-score, immunosuppressive therapy, multiorgan failure, severe dengue

## Abstract

Background: Dengue virus infection is a growing global health concern, especially in tropical and subtropical regions. Dengue fever is commonly self-limiting, but sometimes, rare complications such as hemophagocytic lymphohistiocytosis (HLH) may occur, leading to fatal outcomes. HLH is a rare, hyperinflammatory syndrome marked by excessive immune activation, which can result in multiorgan failure and death. The relationship between dengue and HLH, particularly in adults, is not well understood, and early diagnosis and treatment remain critical for improving outcomes.

Method: A total of 15 adult patients with severe dengue were admitted to a tertiary care ICU setting in North India from September to December 2023. Out of 15 patients, seven were diagnosed with dengue-associated HLH based on H-Score, and bone marrow aspiration was done in six patients. In this retrospective case series, we reviewed their clinical characteristics, laboratory findings, treatment regimens, and outcomes.

Results: The median age of patients was 34 years, with female predominance. The Acute Physiology and Chronic Health Evaluation (APACHE) II score upon admission had a median value of 18, while the Sequential Organ Failure Assessment (SOFA) score at the time of HLH diagnosis had a median value of 11. The median day of illness at the time of HLH diagnosis was 23 days. The H-Score had a median value of 241 at the time of HLH diagnosis, supported by bone marrow aspiration, indicating a >99% probability of HLH. The mortality rate was 50%. Aggressive treatment with corticosteroid therapy, intravenous immunoglobulin (IVIG), or a combination of both, along with other supportive care, was given to the patients.

Conclusion: Persistent fever, altered mental status, bicytopenia, elevated triglyceride levels, and markedly elevated serum ferritin levels are important to differentiate dengue-associated HLH from severe dengue. H-Score can assist in the early identification of HLH. Early recognition and timely initiation of HLH-targeted therapy are essential for improving survival rates.

## Introduction

Dengue fever, an infectious disease, is spread through the bite of infected *Aedes *species mosquitoes that are most common in tropical and subtropical countries. The number of dengue patients reported annually to the World Health Organization (WHO) has increased dramatically from 505,430 cases in 2000 to 5.2 million in 2019. The highest number of dengue cases (6.5 million) was recorded in 2023 [1]. Several factors have contributed to increased dengue cases globally, including climate change, urbanization, and inadequate surveillance and control measures. Climate change, particularly increased temperatures and rainfall, creates favorable conditions for mosquito breeding.

According to the WHO 1997 guidelines, dengue patients can be classified into dengue fever (DF), dengue hemorrhagic fever (DHF), and dengue shock syndrome (DSS) [2]. In 2009, the WHO classified dengue patients as dengue without warning signs (D), dengue with warning signs (DWS), and severe dengue (SD) [3]. Warning signs include abdominal pain or tenderness, persistent vomiting, clinical fluid accumulation, mucosal bleeding, lethargy or restlessness, liver enlargement of >2 cm, and laboratory findings of increasing hematocrit concurrent with rapid decrease in platelet count.

A rare but possibly fatal dengue infection consequence, termed hemophagocytic syndrome, is characterized by marked hyperinflammation, uncontrollably proliferating activated lymphocytes, persistent fever, pancytopenia, jaundice, and hepatosplenomegaly [4]. Hemophagocytic lymphohistiocytosis (HLH) is a subtype of hemophagocytic syndrome. HLH refers to the involvement of both histiocytes and lymphocytes. HLH is characterized pathologically by an improper activation of macrophages in the bone marrow, which leads to blood cell phagocytosis and excessive generation of pro-inflammatory cytokines [5]. Furthermore, because HLH and dengue share many signs and symptoms, including hepatosplenomegaly, leukopenia, thrombocytopenia (together labelled as bicytopenia), and transaminitis, the HLH syndrome is probably underdiagnosed in many dengue-endemic locations. This hidden disorder could contribute to high morbidity and mortality [6].

Early detection of hemophagocytic syndrome in dengue patients may allow better outcomes in patients and early initiation of directed treatment for HLH. A review of the literature suggests a paucity of studies on dengue-associated HLH. To the best of our knowledge, only a few case reports have been published [7-10]. This study analyzes the clinical characteristics, laboratory findings, and management of dengue-associated HLH along with their outcome in adult patients admitted to an intensive care unit (ICU).

## Materials and methods

Study design, setting, and patients

This was a retrospective study of adult patients (age > 18 years) with severe dengue who were admitted to the 30-bed tertiary care ICU setting in North India from September to December 2023. Diagnosis of dengue infection was made based on clinical presentation and laboratory findings, which included antigen detection (NS1), IgM, and IgG antibody detection. Severe dengue was defined according to the WHO 2009 guidelines [2] as plasma leakage leading to shock or fluid accumulation with respiratory distress, presence of severe bleeding and severe organ involvement of (a) liver with elevated liver enzymes (aspartate aminotransferase (AST) and/or alanine aminotransferase (ALT) ≥1000 U/L), (b) impaired consciousness and/ or (c) involvement of heart and other organs.

Diagnosis of dengue-associated secondary HLH

The diagnosis of dengue-associated secondary HLH was made based on a combination of clinical suspicion and laboratory findings and supported by bone marrow biopsy. The probability of HLH was estimated using H-Score (Table 1) [11]. According to Fardet et al. [11], the cut-off value for the H-Score was 169, which corresponded to a sensitivity of 93%, a specificity of 86%, and an accurate classification of 90% of the patients. However, an H-score of 169 points corresponds to only a 40-54% probability of HLH. Therefore, to avoid bias, we excluded one severe dengue patient whose H-Score was >169 but did not undergo bone marrow aspiration to support hemophagocytosis. In bone marrow aspirates from patients with HLH, the presence of hemophagocytosis (macrophages engulf blood cells) was a key characteristic along with histiocytic hyperplasia and the presence of cytotoxic T-cells.

**Table 1:** H-Score parameters and scoring criteria for the diagnosis of HLH HLH: hemophagocytic lymphohistiocytosis Known underlying immunosuppression is defined as being HIV positive or receiving long-term immunosuppressive therapy (i.e., glucocorticoids, cyclosporine-A, azathioprine). Cytopenia is a hemoglobin level of ≤9.2 g/L and/or a total leukocyte count ≤5 × 10^9^/L and/or a platelet count ≤110 × 10^9^/L

Parameters (normal range)	No. of points (criteria for scoring)		
Known underlying immunosuppression	0 (no)	18 (yes)	
Temperature (37°C)	0 (<38.4)	33 (38.4–39.4)	49 (>39.4)
Organomegaly (hepatomegaly and splenomegaly)	0 (no)	23 (any one)	38 (both)
Number of cytopenias	0 (1 lineage)	24 (2 lineages)	34 (3 lineages)
Ferritin (<500 μg/L)	0 (<2000)	35 (2000-6000)	50 (>6000)
Triglyceride (<150 mg/dL)	0 (<132.7)	44 (132.7-354)	64 (>354)
Fibrinogen (200-400 mg/dL)	0 (>250)	30 (≤250)	
Aspartate aminotransferase (<40 U/L)	0 (<30)	19 (≥30)	
Hemophagocytosis on bone marrow aspirate	0 (no)	35 (yes)	

Data collection

Data were retrieved manually from the medical files obtained from the hospital record section and investigations were noted from the Hospital Information System (HIS). We collected the data which include patient demographics, ICU severity scores (Acute Physiology and Chronic Health Evaluation (APACHE) II and Sequential Organ Failure Assessment (SOFA) score) and laboratory parameters, including complete blood count, AST, ferritin, fibrinogen, triglyceride, and lactate dehydrogenase (LDH). We also recorded the presence of fever, hepatosplenomegaly, H-score variables, hemophagocytosis in the bone marrow aspirate, H-score, HLH-specific treatment therapy, need for additional therapy (including dialysis, ventilatory support, vasopressor support, blood product transfusion, and antibiotics), and clinical outcomes.

## Results

A total of 15 adult patients with severe dengue were admitted to the ICU from September to December 2023. Out of 15 severe dengue patients, seven were diagnosed with dengue-associated secondary HLH based on the H-Score, but bone marrow aspiration was done in six patients. Therefore, one patient was excluded as he did not undergo bone marrow aspiration to support the diagnosis of hemophagocytosis, although his H-Score was 176.

The demographic information, ICU severity score, clinical presentations, and laboratory findings of all six dengue-associated HLH patients are shown in Table 2. The median age of patients was 34 years, with female predominance. The median body mass index was 23.7 kg/m^2^. The APACHE II score upon admission had a median value of 18, while the SOFA score upon admission had a median value of 10, and the SOFA score at the time of HLH diagnosis had a median value of 11. The median day of illness at the time of HLH diagnosis was 23 days. The applications of H-Score, given treatment, and outcome are shown in Table 3. The H-Score had a median value of 241 at the time of HLH diagnosis, supported with bone marrow biopsy, indicating a >99% probability of HLH. All the patients were healthy without any comorbidity except one patient who had rheumatoid arthritis controlled on steroids (immunocompromised). The mortality rate was 50%, i.e., three out of six patients succumbed to the illness.

**Table 2:** Demographics, ICU severity score, clinical presentations, and laboratory findings at time of diagnosis of dengue-associated HLH patients BMI: body mass index; APACHE II: Acute Physiology and Chronic Health Evaluation; SOFA: Sequential Organ Failure Assessment; +ve: positive; LDH:  lactate dehydrogenase; PCT: procalcitonin; BM: bone marrow

Parameters (reference range)	Case-1	Case-2	Case-3	Case-4	Case-5	Case-6
Age (year)	43	46	29	25	21	39
Sex (male/female)	F	M	F	F	F	M
BMI (kg/m^2^)	24.6	23.8	22.4	25.3	23.6	23.7
APACHE II (admission)	18	30	18	17	8	18
SOFA (admission)	12	12	14	8	7	8
SOFA (at HLH diagnosis)	16	11	16	9	8	11
Day of illness (at HLH diagnosis)	15	35	16	30	38	16
Dengue serology	NS1 +ve	IgM +ve	NS1 +ve	NS1 +ve	NS1 +ve	NS1 +ve, IgM +ve
Total bilirubin (0.1-1.2 mg/dL)	6.9	3.6	5.3	3.4	31.1	1.7
Serum LDH (120 - 240 IU/L)	1086	882	20340	653	2003	1135
Serum sodium (135 - 145mEq/L)	133	151	134	153	120	129
Consciousness	Altered	Altered	Altered	Altered	Altered	Altered
Ventilator support	Invasive	Invasive	Invasive	Invasive	Non-Invasive	Invasive
Need for a vasopressor	Yes	Yes	Yes	Yes	Yes	Yes
Need of dialysis	Yes	Yes	Yes	Yes	Yes	Yes
Blood product transfusion	Yes	Yes	Yes	Yes	Yes	Yes
PCT (<0.2 ng/mL)	17.01	0.59	0.21	1.67	17.71	1.3
Blood culture	Sterile	Sterile	Sterile	Sterile	Sterile	Sterile
BM culture	Sterile	Sterile	Sterile	Sterile	Sterile	Sterile

**Table 3:** Application of H-Score, given treatment, and outcome of dengue-associated HLH patients MTX: methotrexate; HCQ: hydroxychloroquine; °C: Celsius; TLC: total leukocyte count; AST: aspartate aminotransferase; HP: hemophagocytosis; HLH: hemophagocytic lymphohistiocytosis; IVIG: intravenous immunoglobulin

Parameters (normal range)	Case-1	Case-2	Case-3	Case-4	Case-5	Case-6
Known immunosuppression	No	Yes (MTX, HCQ)	No	No	No	No
Temperature (37◦C)	39.2	38.6	39.1	39.6	38.6	39.1
Hepatomegaly	Yes	No	No	No	Yes	Yes
Splenomegaly	No	No	No	No	Yes	No
Number of cytopenias	Bicytopenia	Bicytopenia	Pancytopenia	Pancytopenia	Pancytopenia	Bicytopenia
Hemoglobin (12-16 gm/dL)	5.6	7.3	8.4	6.7	8.7	10.1
TLC (4-11 x 10^9^/L)	21.98	6.21	3.16	3.77	4.37	3.92
Platelets (150-450 x 10^9^/L)	47	10	26	9	85	7
Ferritin (<500 ng/ml)	26752	3116	69,484	882	20815	2760
Triglyceride (<150 mg/dl)	442	130	731	180	813	374
Fibrinogen (200-400 mg/dl)	295	89	105	302	58	256
AST (<40U/L)	734	215	64	284	450	186
Bone marrow aspirate	Increased histiocytes with significant HP	Increased histiocytes with occasional HP	Increased histiocytes with significant HP	Increased histiocytes with significant HP	Increased histiocytes with occasional HP	Increased histiocytes with significant HP
H-Score	248	194	265	181	303	233
Probability of	>99%	80-88%	>99%	70-80%	>99%	98-99%
Treatment	Antibiotic	Dexa., IVIG, antibiotic	Antibiotic	IVIG, dexa., antibiotic	Dexa., antibiotic	Dexa., antibiotic
Outcome	Death	Death	Death	Discharge	Discharge	Discharge

Case 1

**Figure 1:**
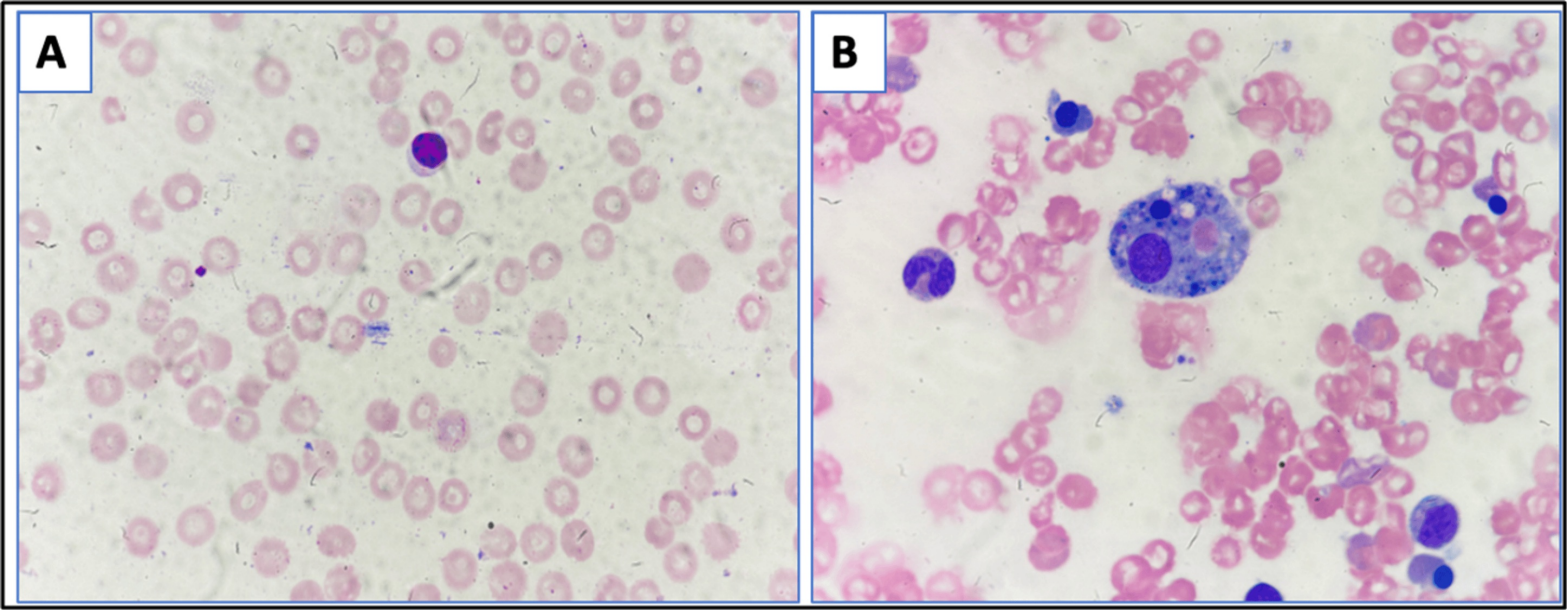
(A) Peripheral smear shows pancytopenia. (B) Bone marrow aspirate showing hypocellular marrow with increased histocytes with hemophagocytosis

A 43-year-old female patient presented with low-grade fever associated with chills, headache, and severe body aches. The patient had a history of travel. Physical findings included subconjunctival hemorrhage. Initial lab investigations showed leukopenia (3.5 x 10^9^/L ), thrombocytopenia (51 x 10^9^/L), hemoglobin (11.8 g/dL), and dengue NS1 positive. On hospital admission day 8, the patient developed altered sensorium and respiratory distress, requiring intubation and mechanical ventilation. The patient continued to have a fever. Further investigations showed bicytopenia, organomegaly, ferritin 26752 ng/mL, fibrinogen 295 mg/dL, triglycerides 442 mg/dL, and AST 734 U/L, raising the suspicion of secondary HLH. Bone marrow aspiration (Figure 1) showed increased histiocytosis with hemophagocytosis, and the H-score was 248 with >99% probability. The patient progressively developed hypotension and was started on high-dose vasopressor support. She also developed anuria, for which dialysis support was given, but because of rapid worsening in hemodynamics, she succumbed to her illness.

Case 2

A 46-year-old male patient with a known case of rheumatoid arthritis on methotrexate and hydroxychloroquine and known hypertension developed low-grade fever with generalized body aches associated with vomiting and loose stools. Initial lab investigations showed leukopenia, thrombocytopenia, and dengue IgM was positive. The patient continued to have a fever and was intubated during the course because of decreased saturation and altered sensorium. HLH workup and bone marrow aspiration were done, which showed an H-Score of 194 with 80-88% probability. He was initially treated with dexamethasone. On the 4th day of dexamethasone therapy, intravenous immunoglobulin (IVIG) 1.6 gm/kg over three days was also added because of a worsening in clinical status. He also developed anuria, for which dialysis support was given. Finally, the patient succumbed to his illness with worsening hemodynamics and organ failures.

Case 3

A 29-year-old female patient with a recent history of lower segment caesarean section (LSCS) developed a fever after two weeks of LSCS. Fever was associated with abdominal pain and vomiting. The patient also had bleeding from the nose. Initial investigation showed thrombocytopenia and dengue NS1 positive. On day 26, the patient was admitted to our hospital and was intubated because of altered sensorium and respiratory distress. She also required vasopressor support. The patient continued to have a fever with TLC 3.0 x 10^9^/L, platelets 26 x 10^9^/L, fibrinogen 105 mg/dL, and procalcitonin 0.21 ng/mL with an H-score of 265 with >99% probability. During the course, there was a rapid worsening of shock, and she succumbed to her illness.

Case 4

A 25-year-old female patient presented with a high-grade fever associated with chills, body aches, and vomiting. Physical examination showed petechial rashes on the upper limbs and chest. She was positive for dengue NS1 antigen. Initial lab investigations showed thrombocytopenia. On day 8 of illness, she was intubated because of altered sensorium. During the course, the patient continued to have a high-grade fever with bicytopenia. A bone marrow aspirate was done, which showed features of increased histiocytes with hemophagocytosis. Initially, she was treated with IVIG (1.6 gm/kg over three days) therapy because of the presence of gastrointestinal bleeding. On day 3 of IVIG, dexamethasone (10mg/m^2^/day) intravenous was added later on, changed to oral, and was tapered gradually. During the ICU course, she also required dialysis. Gradually, the patient improved and was discharged from the hospital.

Case 5

A 21-year-old female patient presented with high-grade fever associated with chills and rigors, generalized weakness, and vomiting. Physical examination includes multiple rashes all over the body, sparing the face, palms, and soles. Lab investigations showed NS1 antigen positive. During the course, the patient had altered sensorium and persistent fever with pancytopenia, for which HLH workup was done and showed a >99% probability. The patient was treated with dexamethasone, and it was tapered gradually. During her hospital stay, she also required noninvasive ventilation support. Gradually, the patient recovered and was discharged from the hospital.

Case 6

A 39-year-old male patient presented with fever associated with chills and rigors, generalized weakness, breathing difficulty, and altered sensorium. Lab investigations showed thrombocytopenia with NS1 and IgM positive. On day 7 of illness, he was intubated because of respiratory distress and altered sensorium. MRI brain showed bilateral multifocal parenchymal microhemorrhages. On day 16 of illness, HLH workup was done and showed 98-99% probability of HLH and treated with dexamethasone, and it was tapered gradually. During the ICU course, he also required dialysis for renal failure. Gradually, the patient recovered and was discharged from the hospital.

## Discussion

Dengue-associated HLH is a rare and life-threatening medical condition characterized by excessive immune activation, posing a significant threat to health. The primary group affected consists of infants, spanning from birth to 18 months old. Nevertheless, the condition can also impact individuals in the pediatric and adult age groups. There are no differences in clinical presentation across different age groups, but the case fatality rate is higher in adult patients [12]. The initial manifestation of HLH may resemble the symptoms commonly associated with other typical infections. Fever and multisystem involvement are the hallmarks of the disease [13]. In our study, fever was persistent in all dengue-associated HLH cases along with multisystem involvement. Limited case reports in adults, even a previous large study [10], have reported secondary HLH in eight dengue patients. Five out of eight patients who underwent bone marrow aspiration had evidence of hemophagocytosis. Delay in recovery from the expected course of dengue should be an alert for the clinician to suspect a diagnosis of HLH.

The underlying mechanisms of HLH are not thoroughly understood. One suggested theory is that an elevated count of activated macrophages arises due to improper proliferation and activation of T-cells. These activated macrophages lose their ability to eliminate intracellular macrophages. The HLH subtypes involve perforin and NK cells. In the scenario of perforin deficiency, the defense mechanisms against intracellular pathogens are compromised. Conversely, reduced NK cell activity results in heightened T-cell activation, leading to the production of abundant cytokines like tumor necrosis factor alpha (TNF-α), interferon gamma (IFN-γ), and granulocyte-macrophage colony-stimulating factor (GM-CSF) [14]. This sequence of events culminates in persistent macrophage activation leading to multi-organ failure, as was seen in our cases.

In the past, the diagnosis of HLH relied on the presence of fever, splenomegaly, cytopenia, hypertriglyceridemia, hypofibrinogenemia, and hemophagocytosis. However, in 2004, the Histiocyte Society introduced three additional criteria: low or absent NK cell activity, hyperferritinemia, and elevated soluble interleukin-2 receptor levels. HLH-2004 diagnostic requirement involves the fulfillment of five out of the preceding eight criteria [15-17]. In 2014, Fardet et al. developed and validated the H-Score for the diagnosis of reactive hemophagocytic syndrome [11]. The cut-off value for the H-Score was 169, which corresponded to a sensitivity of 93%, a specificity of 86%, and an accurate classification of 90% of the patients. However, an H-score of 169 points corresponds to only a 40-54% probability of HLH. HLH has been categorized into two types: primary HLH is a rare, linked to an underlying genetic condition, and secondary HLH is more common, associated with infectious, malignant, rheumatologic, or other secondary factors. Infection can also serve as a trigger for primary HLH [15]. Secondary HLH cases are mostly due to infectious causes, leading to infection-associated hemophagocytic syndrome (IAHS). Epstein-Barr virus (EBV) stands out as the most prevalent cause. CMV, parvovirus, herpes simplex virus (HSV), varicella-zoster virus (VZV), measles virus, human herpesvirus 8, H1N1 influenza virus, parechovirus, and HIV are all implicated in the development of secondary HLH. Additionally, tuberculosis, enteric fever, malignancies (leukemias and lymphomas), and autoimmune conditions (systemic lupus erythematosus (SLE), adult-onset Still's disease, and juvenile rheumatoid arthritis) can also lead to secondary HLH [15-18].

In EBV-HLH and dengue-induced-HLH, T-cells become infected. Moreover, in dengue, T-cells contribute to viral replication and the release of viral particles. Therefore, the rationale is to treat dengue-induced HLH similarly to EBV-HLH. Corticosteroids and etoposide are employed to diminish lymphocytes. Etoposide has demonstrated its efficacy as a crucial component in the treatment of both primary and secondary HLH [19-22]. The precise mechanism by which etoposide operates in hyperinflammation is not thoroughly comprehended. However, its role in selectively eliminating activated T-cells and decreasing inflammatory cytokines contributes to the amelioration of HLH conditions [23].

The use of dexamethasone (10 mg/m^2^/day) in HLH treatment is also deemed highly advantageous [24]. We also used dexamethasone in four out of six patients. In two patients, we were unable to add specific therapy because of rapid worsening leading to death. Elevated levels of ALT, AST, creatinine, LDH, and ferritin persist notably in severely affected patients. Elevated AST and maximum ferritin levels are associated with increased mortality. In our study, three patients died out of six patients. Clinicians should contemplate the possibility of HLH in patients with dengue infection if they note persistent fever, altered mental state, cytopenia with organ involvement, and particularly if ferritin exceeds 10,000 μg/L [25].

Treating dengue-associated HLH involves addressing the underlying cause, yet the anti-inflammatory properties of pulse dose glucocorticoids, such as methylprednisolone or dexamethasone, can also be employed. IVIG may be used up to 1.6 gm/kg in split doses over 2-3 days independently or in conjunction with dexamethasone or methylprednisolone [26]. We also used it on two patients, out of which one patient survived. IVIG has anti-inflammatory potential by inhibiting complement activation, blocking antibody Fc fragments and macrophage Fc receptors, and neutralizing cytokines [27]. Interestingly, anakinra, an interleukin-1 inhibitor, has shown promising results in treating secondary HLH in sepsis patients [28], with or without dexamethasone, offering the advantage of deliberately avoiding the use of etoposide. Diagnosing dengue-associated HLH is challenging but crucial for recognition, as it is associated with improved treatment options, especially if introduced early in the course of illness.

In our cases, we did not identify the dengue viral serotype. Prospective studies with large sample sizes are needed to better identify the relation between dengue-associated HLH and viral serotypes. It will increase our understanding of the disease course and prognosis and help in the more effective and timely management of secondary HLH. Limitations of this case series include its retrospective nature, small case series, absence of monitoring of inflammatory markers, and lack of follow-up for organ dysfunctions.

## Conclusions

Severe dengue with delayed recovery from primary infection should be an alert for clinician for secondary HLH. Persistent fever, altered mentation, bicytopenia, elevated triglyceride levels, and markedly elevated serum ferritin levels differentiate dengue-associated HLH from severe dengue. Early recognition and initiation of HLH-directed therapy can be crucial for the successful treatment of dengue fever complicated by HLH. 
